# In Vitro and In Vivo Improvement of Islet Quality and Transplantation Successes following Islet Treatment with Biomaterials in Diabetic Rats

**DOI:** 10.1155/2023/1399917

**Published:** 2023-05-24

**Authors:** Marzieh Nemati, Zahra Ebrahimi, Narges Karbalaei, Sanaz Dastghaib, Sara Khakshournia, Mojtaba Sargazi

**Affiliations:** ^1^Endocrinology and Metabolism Research Center, Shiraz University of Medical Sciences, Shiraz, Iran; ^2^Department of Biology, Science and Research Branch, Islamic Azad University, Tehran, Iran; ^3^Department of physiology, Shiraz University of Medical Sciences, Shiraz, Iran; ^4^Histomorphometry and Stereology Research Center, Shiraz University of Medical Sciences, Shiraz, Iran; ^5^Authophagy Research Center, Shiraz University of Medical Sciences, Shiraz, Iran; ^6^Department of Biochemistry, Shiraz University of Medical Science, Shiraz, Iran

## Abstract

**Background:**

Loss of islet survival and function, caused by native niche disruption and oxidative stress induction during mechanical and enzymatic isolation, limits the effectiveness of islet transplantation. Reconstitution of islet microenvironment, vascularization, and decreased oxidative stress with biomaterials may improve islet quality and graft outcomes. We investigated effects of two biomaterials, platelet-rich plasma and pancreatic islets homogenate combination on islet recovery and quality by evaluating in vitro islet survival, secretory function, and oxidative stress parameters and assessing in vivo transplantation outcomes.

**Methods:**

In vitro, islet viability and secretory function of isolated islets were assessed after 24 h and 72 h incubation with biomaterials. Also, oxidative stress markers were measured once after isolation and 24 h after incubation with biomaterials. For evaluating in vivo effects, cultured islets for 24 h were transplanted into subscapular space of diabetic rat kidney, and outcomes were analyzed by measuring serum glucose and insulin concentrations, glucose tolerance test, level of oxidative parameters, and pancreatic gene expression.

**Results:**

Treating islets with biomaterials significantly increased their viability and secretory function, reduced MDA level, and elevate SOD and CAT activity. Decreased level of glucose and MDA improved insulin level, increased SOD activity, and also enhanced pdx_1_ and insulin gene expression in diabetic rats after islet transplantation.

**Conclusions:**

Biomaterials used in the present study should be consider as beneficial materials for increasing islet transplantation outcome. These materials may hamper transplantation limitation to some extent.

## 1. Introduction

Pancreatic islet transplantation therapy restores glucose homeostasis and recommended as an effective and alternative treatment method for diabetes [1–4]; however, success rate of transplantation such as viability and function is limited by [5–7] several factor, including niche disruption, insufficient revascularization, oxidative stress, and extracellular matrix- (ECM-) islet cell interaction impairment [8] that happens during islet isolation process.

Considering that the interaction between beta cells and ECM is necessary for the growth, survival, and secretory function of these cells [9], it seems that restoring natural islet microenvironment and decreasing oxidative stress may be a potential choice for improving graft survival and ameliorating transplantation outcomes.

In line with this strategy, diverse materials such as ECM proteins [10–12], Matrigel [13], small intestinal submucosa (SIS) (contains various extracellular matrix and growth factors) [12, 14], and mesenchymal stem cells [14] have been used. Several studies showed that ECM supplementation by imitating the biochemical constitution could improve islet transplantation [15–17]. Oxidative stress during islet isolation and after islet transplantation is the other main factor that decreases the islets' survival and function. So in recent years, the use of antioxidant before [18–20] and after [21–24] transplantation in order to optimize islet function and survival is an interested area of investigation. However, loss of transplanted islet survival and function is still significant limitation that should be resolved.

With a goal to ameliorate the success of islet transplantation, we investigated the effects of using of two biomaterials (PRP&PIH) which are rich sources of ECM and growth factors. Previous studies have demonstrated the efficacy of PRP and PIH alone.

Platelet-rich plasma (PRP) contains varied growth factors activating intracellular signaling pathways and induces protein production which is necessary for processes such as proliferation, differentiation, collagen, and matrix production [25]. The other advantages of PRP include easy handling and lack of immune response in recipient [26, 27], which made PRP safe to be used successfully in different fields. In addition, antioxidant capacity of PRP or its growth factors such as insulin-like growth factor (IGF-1) were shown in several studies [28–30]. Also, in our previous investigation, PRP could boost islet survival, function, and transplantation outcome [31].

Pancreatic islet homogenate (PIH) is another biomaterial that should be regarded as a suitable candidate for enhancing islet quality and transplantation outcome because it includes various proteins and growth factors such as collagen, laminin, fibronectin, VEGF-A, and HGF which have a variety range of beneficial effects on islets [32, 33]. Also, its other advantages include more adaptable in texture, immunity, and cellular mechanisms compared with other synthetic materials and more reasonable due to unsuitable isolated islets for transplantation were homogenated and used as supplementary biomaterial in transplantation. In addition, antioxidant capacity of some ECM proteins was demonstrated in several studies [34, 35]. Our previous study showed positive effects of PIH on islet quality and transplantation outcome [36].

The aim of the present study is whether using these biomaterial combinations, with higher concentration of growth factors and ECM, can improve the islet recovery, the quality, and the success of transplantation by mimicking the biochemical composition of the islet normal niche and also by reducing oxidative stress.

## 2. Materials and Methods

### 2.1. Ethical Statement

Male Sprague-Dawley rats (260-280 g) aged 12-13 weeks were purchased from the stock of bred in animal facility of Research Institute of Shiraz University of Medical Sciences (Shiraz, Iran) and used as donors and diabetic islet recipients. Animals were housed under standard lighting conditions (12-hour light/dark cycle) at temperature of 22 ± 2°C and relative humidity of 23 ± 5% with free access to water and food.

### 2.2. Study Design

In vitro experiments, isolated islets from Sprague-Dawley rats were divided into four groups (10 equivalent islets in each group): islets were cultured and incubated for different times 24 h and 72 h [14] in 1 ml RPMI without any treatment (Control-Islet) or in 900 *μ*l RPMI+100 *μ*l PRP (10%, plt: 1500 × 10^3^/*μ*l) (PRP-Islet) or in 900 *μ*l RPMI+100 *μ*l PIH (10%, pro: 100 *μ*g) (PIH-Islet) or in 800 *μ*l RPMI+100 *μ*l PRP (10%, plt: 1500 × 103/*μ*l)+100 *μ*l PIH (10%, pro: 100 *μ*g) (PRP&PIH-Islet); then, islet quality was analyzed by assessing the viability and insulin release in response to basic (5 Mm) and stimulated (11 Mm) glucose concentration and assessing insulin content. Also, oxidative stress markers (MDA, SOD, and CAT) were measured at once after isolation and 24 h after incubation.

In vivo experiments, 42 rats were randomly allocated into six groups as follows (7 rats/group): control (untreated rats), diabetic (diabetic control rats), IT (diabetic rats were transplanted with islet only), IT-PRP (diabetic rats were transplanted with PRP treated islet), IT-PIH (diabetic rats were transplanted with PIH-treated islet), and IT-PRP&PIH (diabetic rats were transplanted with PRP&PIH-treated islet). The diabetic rats received 400 islet equivalents (IEQ) under the left kidney capsule. Blood samples were collected from tail vein on day 0 (day of transplant) and 60-day posttransplantation for assessing serum glucose and insulin concentrations. At the end of experiment, intraperitoneal glucose tolerance tests (IPGTT), pancreatic pdx1, insulin gene expression, and serum oxidative stress parameter (MDA and SOD) assessment were performed. The collected serums were sent to a specialized laboratory to measure the serum concentrations of various parameters. It should be noted that the samples were numbered and the group of animals was not mentioned and was done blindly.

### 2.3. Diabetic Induction

Diabetes was induced by intraperitoneal (i.p.) injection of STZ (65 mg/kg, i.p.; Sigma). Blood was collected from tail vein and blood glucose was determined using a glucometer (Accoutered Plus; Roche, Mannheim, Germany). The rats were included in the study when fasting blood glucose (>350 mg/dl) and accepted as diabetic rats and used as transplant recipient. The animals were excluded if blood glucose levels were lower than 350 mg/dl and if transplanted animals developed ascites after surgery [36].

### 2.4. Platelet-Rich Plasma Preparation

Rats were anesthetized with ketamine and xylene (50/10 mg/kg), whole blood was collected through cardiac puncture and drained into 15 ml centrifuge tube containing anticoagulant (3.2% sodium citrate with 9/1 ratio blood to sodium citrate), and then, PRP was prepared as already reported [31]. For measuring the concentration of three growth factors, we add 50 *μ*l calcium chloride 10% to 1 ml of obtained PRP pool and then incubated for 30 min to clot formation. Clot was centrifuged at 2500 rpm for 20 min in 4 centigrad degree, then supernatant was separated for measuring growth factors concentration. Insulin-like growth factor-1 (IGF-1) was assayed by rat IGF-1 enzyme-linked immunosorbent assay method (ELISA) (Thermo Fisher, USA, sensitivity: 30 pg/ml, assay range: 30.72-7500 pg/ml), TGT-*β* was assayed by rat TGT-*β* ELISA method (Thermo Fisher, USA, sensitivity: 7.8 pg/ml, assay range: 31.25-2000 pg/ml), VEGF was assayed by rat VEGF ELISA method (Thermo Fisher, USA, sensitivity: 2 pg/ml, assay range: 0.82-200 pg/ml), and HGF was assayed by rat HGF ELISA method (Thermo Fisher, USA, sensitivity: 2 pg/ml, assay range: 0.82-200 pg/ml) (Table 1).

### 2.5. Islet Isolation

**Figure 1:**
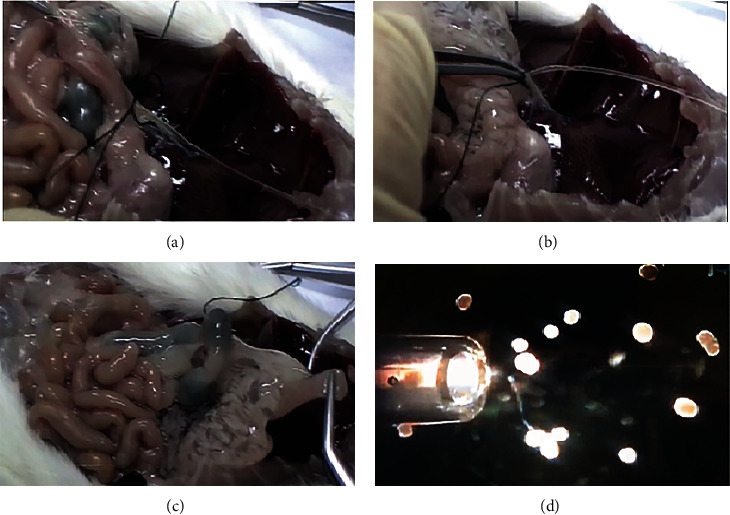
The isolated islets from rats. Common bile duct cannulation and fixing it (a). Clamp the end of common bile duct to the intestine and injecting collagenase (b). Pancreas was separated from its surrounding tissues (intestine, spleen, stomach) (c). Islet hand picking under stereomicroscope (d).

Pancreatic islet isolation from rats was performed after overnight fasting. After anesthesia with ketamine/xylazine (50/10 mg/kg) and laparotomy, the islets were isolated by the collagenase method (Safayee et al. 2016). The pancreas was dissected and entrance of common bile duct to the duodenum was clamped, the bile duct was cannulated with a polyethylene catheter (Portex Intravenous Cannula 2.5 F, 0.75 mm OD, Kent, UK), and 10 ml ice-cold Hanks' balanced salt solution containing collagenase P (Roche, Cat. # 11 213 865 001, Mannheim, Germany, 0.5 mg/ml) was gently perfused into the duct. The inflated pancreas was removed, cleaned from nonpancreatic tissue, and incubated for 17 min at 37°C in water bath. After washing, the islets were handpicked under a stereomicroscope (Blue Light stereomicroscope, La Habra, CA) and cultured in 1 ml RPMI-1640 media [29] supplemented with or without PRP and PIH and incubated at 37°C in 5% CO_2_ and 95% air for 24 hours (Figure 1).

### 2.6. Pancreatic Islet Homogenate Preparation

After adding lysis buffer to unsuitable pancreatic isolated islet for transplantation, they were homogenated and then PIH pool was prepared as already reported [36]. IGF-1, TGF-*β*, VEGF, HGF, and collagen I were measured by rat collagen I ELISA method (Abcam, UK, sensitivity: 0.938 ng/ml, assay range: 1.563-100 ng/ml) and PIH was measured (Table 1).

### 2.7. Culture of Isolated Islets

Isolated islets were cultured in 1 ml RPMI-1640 media (RPMI-1640 containing static or low concentration of glucose (5 Mm) or high and stimulate glucose concentration (11 Mm), 10% FBS, 0.5% BSA (Sigma-Aldrich) and 1% pen strep)) that are supplemented without or with PRP, PIH, or their combination and incubated at 37°C in 5% CO_2_ and 95% air for 24 hours and 72 h.

### 2.8. Islet Viability and Function

After 24 and 72 h incubation, islet quality was determined by evaluating islet viability and secretory function. Islet cell viability was evaluated by Annexin V and propidium iodide (PI) staining and defined as percentage of viable cells (stained by Annexin V, Life Technologies Japan, Tokyo, Japan) per total number of viable and dead cells (stained by propidium iodide, Sigma-Aldrich) at time point (viability rate% = numbers viable cells (green)/total number of viable and dead cells (green + red) × 100) [31].

Insulin concentration in the culture medium and insulin content (with acid ethanol extraction protocol as already reported) were assessed by rat insulin ELISA assay method (Mercodia and Uppsala, Sweden, detection limit ≤ 0.15 *μ*g/l). Protein was assayed by commercial Thermo Scientific Pierce BCA Protein Assay Kit (Rockford, IL, USA, sensitivity: 5 *μ*g/ml, assay range: 20-200 *μ*g/ml) [31]. Stimulation index (SI) was used to measure the ratio of insulin release in response to high glucose concentration/insulin secretion in response to low glucose level [12].

### 2.9. Islet Transplantation

Aliquots of 400 islet equivalents that are cultured for 24 hours with or without PRP and PIH were aspirated into a polyethylene tubing P-50 (Harvard Apparatus, Holliston, MA, USA) and placed on ice. The recipient animals were anesthetized with ketamine and xylene (50/10 mg/kg), and islets were transplanted under the capsule of left kidney as already reported [31].

### 2.10. Intraperitoneal Glucose Tolerance Test (IPGTT)

IPGTT was performed after animals had been fasted for 16 h. Plasma glucose and insulin levels were measured at 0 (before glucose injection), 30, 60, and 120 min after intraperitoneal injection of 2 g/kg glucose concentration [37].

Serum glucose and insulin concentrations were measured by the glucose oxidase method (Pars Azmoon Co., Tehran, Iran), and plasma insulin concentrations were measured by the ultrasensitive rat insulin ELISA (Mercodia, Sweden, detection limit ≤ 0.15 *μ*g/l). For the calculation of homeostasis model assessment of insulin resistance index (HOMA-IR), the following formula is used [31]: fasting glucose (mg/dl) × fasting insulin (ng/ml)/22 : 5.

### 2.11. Oxidative Stress Markers

Serum and in vitro malondialdehyde (MDA) levels of cultured islet (previously treated with biomaterials) were measured manually by thiobarbituric acid reactive substance (TBAR) method. Serum and in vitro superoxide dismutase (SOD) activity and in vitro catalase (CAT) activity were computed by commercial assay kits (ZellBio GmbH, Ulm, Germany) using colorimetrical method (SOD, sensitivity: 0.044, assay range: 5-100 U/ml, and CAT, sensitivity: 0.5 U/ml, assay renge: 1-100 U/ml) [31, 38] .

### 2.12. Reverse Transcription Polymerase Chain Reaction Analysis (RT-PCR)

Dissected pancreatic tissues from transplanted rats immersed into RNA later solution (Ambion, AM7021, and Austin, USA) for 24 hours and kept in -80°C. Total RNA was extracted with TriPure Isolation Reagent (Roche, Germany) according to the manufacturer's instructions. The cDNA was synthesized from 1 *μ*g of total RNA by using RevertAid First Strand cDNA Synthesis Kit (Fermentas, Germany) with random hexamer and oligo dT primers following the manufacturer's protocol. PCR was performed in an ABI 7300 PCR System (Applied Biosystems Co., Carlsbad, CA, USA) with different primers (*β*-actin forward: 5′-CCACACCCGCCACCAGTTCG-3′ and reverse: 5′-CTAGGGCGGCCCACGATGGA-3′; Pdx1 forward: 5′-GCGTTCATCTCCCTTTCCC and reverse: 3′-GGTCCTCTTATTCTCCTCCG; and insulin: 5′-AGC AAG CAG GTC ATT GTT CC and reverse: 3′-TTG CGG GTC CTC CAC TTC 209). Real-time PCR was performed by using SYBR-Green PCR Master Mix kit (TaKaRa, Japan) in ABI real-time PCR 7500 system. Data were analyzed by using 7500 Software v 2.0.1. Relative expression level of insulin and Pdx1 genes was calculated by 2-*ΔΔ*CT formula. *β*-Actin was considered as an internal control [14].

### 2.13. Statistical Analysis

Statistical data analysis was performed using GraphPad Prism software version 6.0 (GraphPad Software, La Jolla, CA, USA) and presented as means ± SEM. One-way ANOVA (post hoc: Tukey) was used *for* multiple comparisons and repeated measure two-way ANOVA (post hoc: Bonferroni) was used to *analyze* plasma glucose and insulin concentrations during IPGTT. A value of *p* < 0.05 was considered statistically significant.

## 3. Results

### 3.1. Islet Quality Assessment

**Figure 2:**
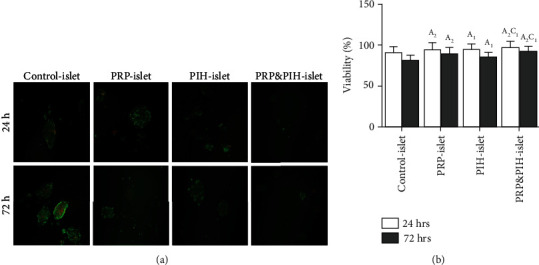
Effect of PRP&PIH on the islet cell viability. For determination of islet cell viability, AV/PI staining was performed. The live islet cells were stained green and dead cells stained red. Magnification: 100x (a). The barograph represented the percentage of the viability of the islets that was defined as the ratio of viable cells to total viable and dead cells in each islet (b). Data are presented as mean ± SEM (*n* = 10). Data are presented as mean ± SEM (*n* = 7). ^A^Statistically significant differences compared to Control-Islet. ^B^Statistically significant differences compared to PRP-Islet. ^C^Statistically significant differences compared to PIH-Islet. ^1^*p* < 0.05, ^2^*p* < 0.01, ^3^*p* < 0.001, and ^4^*p* < 0.0001. Control-Islet: untreated islet; PRP-Islet: islet treated with PRP; PIH-Islet: islet treated with PIH; PRP&PIH-Islet: islet treated with PRP&PIH.

Islet viability percentage of all treated islet groups in both 24 h and 72 h after incubation was significantly higher than control islet that was more markedly higher in the PRP-Islet and PRP&PIH-Islet groups than PIH-Islet. In comparison between treated islets, viability rate was significantly higher in PRP&PIH than the PIH-Islet group in both 24 h and 72 h after incubation. Also, comparison between two times showed that there were no significant differences between two times in all groups (Figure 2).

**Figure 3:**
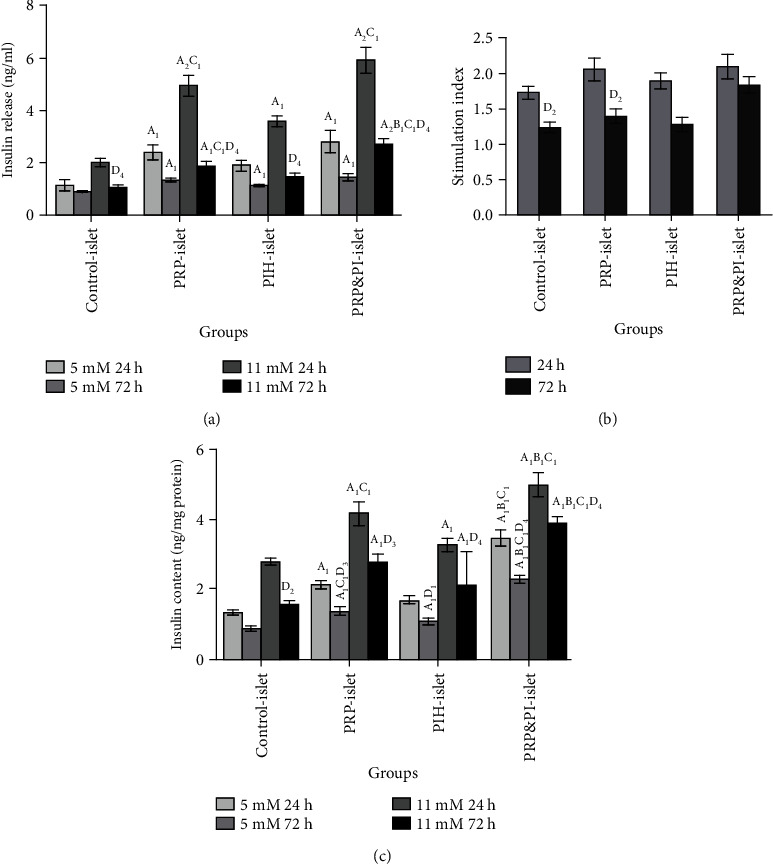
Effect of PRP&PIH on insulin release, stimulation index, and insulin content. Data are presented as mean ± SEM from 8 batches of ten islets from 6 rats. ^A^Statistically significant differences compared to control in the same concentration and time. ^B^Statistically significant differences compared to PRP-Islet group in the same concentration and time. ^C^Statistically significant differences compared to PIH-Islet group in the same concentration and time. ^D^Statistically significant differences compared to in the same concentration and group and different time. ^1^*p* < 0.05, ^2^*p* < 0.01, ^3^*p* < 0.001, and ^4^*p* < 0.0001. Control-Islet: untreated islet; PRP-Islet: islet treated with PRP; PIH-Islet: islet treated with PIH; PRP&PIH-Islet: islet treated with PRP&PIH.

As shown in Figure 3, insulin release of all treated islet groups at 24 h and 72 h in response to both basic and stimulated glucose concentrations (except PIH-Islet in basic level at 24 h and stimulated level in 72 h) was significantly higher than control islet. This elevation in PRP&PIH was more significant than the PIH-Islet group in high glucose concentration after 24 h and 72 h incubation. In addition, the level of insulin secretion in response to high glucose level after 72 h incubation in the PRP&PIH-Islet group was more significant than PRP-Islet and also more markedly higher in PRP-Islet than the PIH-Islet group. Also, comparison between two times showed that insulin release in response to 11 Mm glucose concentration in all groups decreases in 72 h compared to 24 h.

Stimulation index at 24 and 72 h was higher in all treated islet groups compared to Control-Islet but was significant only in PRP&PIH-Islet. Comparison between two times, stimulation index in the control and PRP-Islet groups was significantly decreased; however, this reduction was not significant in the PIH-Islet and PRP&PIH-Islet groups (Figure 3).

Insulin content was higher in all treated islets (except PIH-Islet in low glucose concentration at 24 h after incubation) in both basic and stimulated glucose concentration and both times compared to control islets. Comparison between treated islet showed that at 24 h and 72 h in 5 mM and 11 mM glucose concentration, insulin content was remarkably higher in PRP&PIH-Islet than PRP-Islet and PIH-Islet (Figure 3).

The MDA level in 24 h/once after isolation ratio showed significant decrease in all treated groups, but there was not any change in the control group. At 24 h, all treated islet groups showed significant decrease in MDA level compared to the control group; this reduction was more markedly in PRP&PIH-Islet. Among different treated islets, there was no difference (Table 2).

The SOD activity in 24/once after isolation was significantly increased in all treated islet groups; this elevation was more in the PRP-Islet and PRP&PIH-Islet groups. At 24 h after incubation, this parameter in all treated islet groups was notably higher than the control group. Among treated islets, SOD activity was significantly higher in PRP&PIH-Islet than the PRP-Islet and PIH-Islet groups (Table 3).

CAT activity in 24/once after isolation ratio in all treated groups was markedly higher than the control group. This enhancement was more significant in PRP&PIH-Islet than the PRP-Islet and PIH-Islet groups. At 24 h after incubation, CAT was significantly higher in all treated islets than control islets; this significance was more in PRP&PIH-Islet. Among treated islets, there was markedly higher CAT activity in PRP&PIH-Islet than the PRP-Islet and PIH-Islet groups (Table 3).

### 3.2. Posttransplant Outcomes

#### 3.2.1. Body Weight

At the end of experiment (60-day posttransplantation), body weight except in the IT-PRP&PIH group, in other animals, was significantly lower than the control group; this difference was more in diabetic control animals. In comparison with the diabetic group, animals receiving islet transplantation showed markedly increase in body weight. Among all diabetic transplanted animals, this parameter was higher in treated islet transplantation groups than untreated islet-transplanted animals, but this difference was only significant in IT-PRP&PIH animals. Comparison between diabetic animals receiving treated islets, body weight enhancement was more significant in the IT-PRP&PIH group than IT-PRP and IT-PIH animals. Except the diabetic control group (10.49 percentage of weight loss), there was weight gain % in all experimental groups; however, in the diabetic transplanted groups, it was significantly lower than the control group. In comparison with diabetic group, all transplanted groups showed markedly higher weight gain percentage. Also, the percentage of weight gain in the PRP-Islet and PRP&PIH-Islet groups was notably higher than the IT group. Among the islet-treated transplantation groups, the IT-PRP&PIH group showed significantly higher percentage of weight gain than the PRP-Islet and PIH-Islet groups; this difference was more than the PIH-Islet group. Also, percentage of weight gain in PRP-Islet was significant than PIH-Islet (Table 2).

### 3.3. Serum Glucose and Insulin Concentrations

As shown in Table 4, at the end of experiment, except the IT-PRP and IT-PRP&PIH groups, other animals showed significant higher serum glucose levels compared with the control group. Also, serum glucose concentration in all transplanted animals was markedly lower than diabetic animals. Animal receiving treated islet showed lower mean glucose concentration than that in animals receiving untreated islets. Among the treated islet transplanting groups, this parameter was markedly lower in the IT-PRP&PIH group than animals transplanted with islets treated with only one biomaterial (IT-PRP and IT-PIH groups). The level of glucose in IT-PRP&PIH animals demonstrated normal glycemic status and was less than 115 mg/dl. The glucose concentration in day 60 was significantly lower in all diabetic transplanted animals compared to day 0 in the same group. But this difference was different in the diabetic group, and the glucose level was increased.

At the end of experiment, except the diabetic control group, serum insulin levels were increased following transplantation in all diabetic transplanted groups. Among the islet-transplanted diabetic groups, transplanting with treated islets showed significant increment in insulin concentration compared to animal transplanting with untreated islets. Treatment islet with PRP&PIH combination resulted in greater increase in insulin levels than when only one biomaterial was used alone, as there was no significant difference in this parameter between the IT-PRP&PIH and control groups. This parameter in day 60 was significantly higher in islet-transplanted animals compared to day 0 in the same group. But, this difference was not shown in the diabetic control group (Table 2).

### 3.4. Fasting Serum Glucose and Insulin Concentrations and HOMA-IR

**Figure 4:**
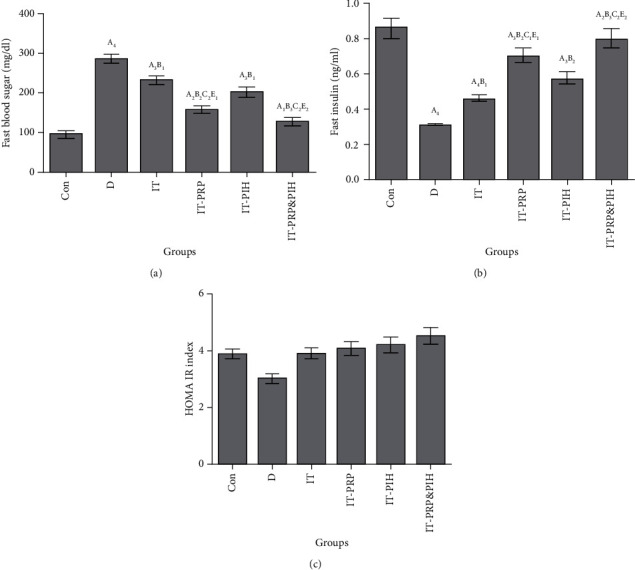
Effect of PRP&PIH on fasting glucose, insulin, and HOMA IR index. Data are presented as mean ± SEM (*n* = 7). ^A^Statistically significant differences compared to control. ^B^Statistically significant differences compared to the D group. ^C^Statistically significant differences compared to the IT group. ^D^Statistically significant differences compared to the IT-PRP group. ^1^*p* < 0.05, ^2^*p* < 0.01, ^3^*p* < 0.001, and ^4^*p* < 0.0001. D: control diabetic rats; IT: islet-transplanted diabetic rats; IT-PRP: islet-transplanted diabetic rats with PRP; IT-PIH: islet-transplanted diabetic rats with PIH; IT-PRP&PIH: islet-transplanted diabetic rats with PRP&PIH.

A significant decrease and increase in fasting glucose and fasting insulin levels, respectively, were observed in the diabetic groups. In comparison with the diabetic group, all transplanted diabetic animals showed significant decrease in fast blood glucose. Also, in all islet-treated transplanted animals, the level of fast glucose concentration was markedly lower than untreated islet transplantation animals. Among the islet-treated groups, IT-PRP&PIH indicated a significant reduction in fasting glucose level versus IT-PIH animals. The level of fasting insulin in all the diabetic group was significantly lower than the control group. This parameter in islet transplanted groups was notably lower than diabetic group, and in animals receiving untreated islets was significantly lower than that in treated islets transplanting groups. Among the islet-treated groups, IT-PRP&PIH indicated a significant elevation in fasting insulin level versus IT-PRP and IT-PIH animals. In HOMA-IR index, there were no significant differences among the experimental groups (Figure 4).

### 3.5. Intraperitoneal Glucose Tolerance Tests

**Figure 5:**
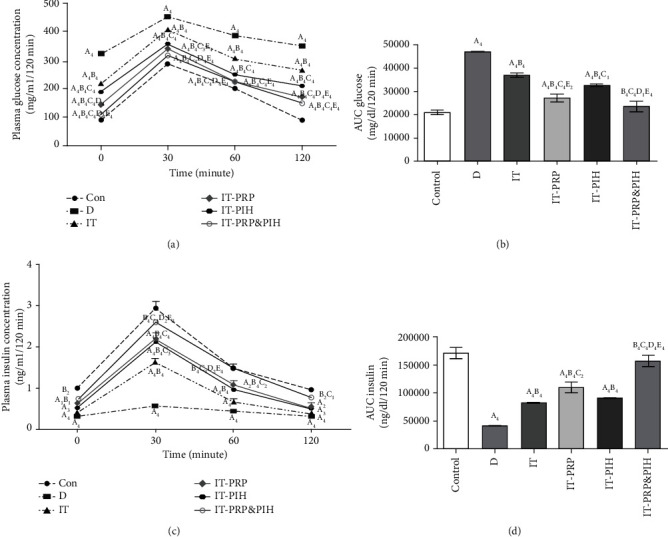
Change in IPGTT serum glucose (a), IPGTT serum insulin concentration (c), and their AUC values (b, d) after islet transplantation. Data are presented as mean ± SEM (*n* = 7). ^A^Statistically significant differences compared to control. ^B^Statistically significant differences compared to the D group. ^C^Statistically significant differences compared to IT group. ^D^Statistically significant differences compared to the IT-PRP group. ^E^Statistically significant differences compared to IT-PIH group. ^1^*p* < 0.05, ^2^*p* < 0.01, ^3^*p* < 0.001, and ^4^*p* < 0.0001. D: control diabetic rats; IT: islet-transplanted diabetic rats; IT-PRP: islet-transplanted diabetic rats with PRP; IT-PIH: islet-transplanted diabetic rats with PIH; IT-PRP&PIH: islet-transplanted diabetic rats with PRP&PIH.

In all the diabetic group, glucose concentrations during glucose tolerance test were significantly higher than the control group. In comparison to D, mean blood glucose levels significantly decreased in diabetic islet-transplanted animals during glucose tolerance test. Among islet-transplanted animal, the treated islet-transplanted groups (IT-PRP, IT-PIH, and IT-PRP&PIH) mean serum glucose concentration was significantly lower than untreated transplanted animals within IPGTT. Also, glucose concentrations in IT-PRP&PIH at time points 0, 30, and 120 min and in IT-PRP at all time points were significantly lower than the IT-PIH group (Figure 5(a)). Plasma glucose area under curve (AUC) in the diabetic group (except combination animal) was significantly higher than the control group. In comparison to diabetic control rats, glucose AUC was decreased remarkably in transplanting animals. The combination group showed significant increase in plasma glucose AUC compared to animal received islet treated with only one biomaterial (Figure 5(b)).

Data of IPGTT showed a significant reduction in mean insulin levels in all diabetic groups at all time points except at 120 min in IT-PRP&PIH, compared to the control group. In comparison to D group, mean insulin levels increased markedly in the diabetic transplanted groups. The mean insulin levels in all treated islet-transplanted groups were significantly higher than untreated islet-transplanted animals. Comparison among islet-treated transplanted groups, animals receiving both biomaterials (IT-PRP&PIH) showed significantly higher mean insulin concentration at 0, 30, and 120 than IT-PRP and also at all point than the IT-PIH groups (Figure 5(c)). Plasma insulin AUC in all the diabetic groups except combination animals was significantly lower than in the control group. Compared to the D group, this parameter was significantly increased in transplanting animals. Plasma insulin AUC in animal receiving both materials was markedly higher than those receiving just one biomaterial (Figure 5(d)).

### 3.6. Pancreatic pdx_1_ and Insulin Gene Expression

**Figure 6:**
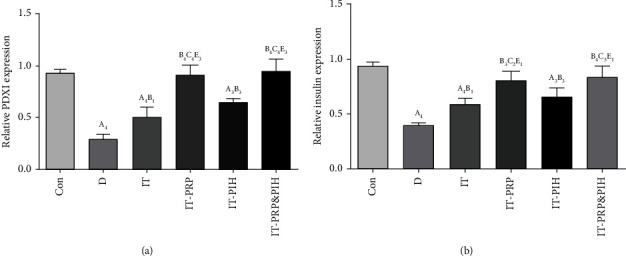
Pdx1 (a) and insulin (b) gene expression levels after islet transplantation. Data are presented as mean ± SEM (*n* = 7). ^A^Statistically significant differences compared to control. ^B^Statistically significant differences compared to the D group. ^C^Statistically significant differences compared to the IT group. ^D^Statistically significant differences compared to the IT-PRP group. ^E^Statistically significant differences compared to the IT-PIH group. ^1^*p* < 0.05, ^2^*p* < 0.01, ^3^*p* < 0.001, and ^4^*p* < 0.0001. D: control diabetic rats; IT: islet-transplanted diabetic rats; IT-PRP: islet-transplanted diabetic rats with PRP; IT-PIH: islet-transplanted diabetic rats with PIH; IT-PRP&PIH: islet-transplanted diabetic rats with PRP&PIH.

As shown in Figures 6(a) and 6(b), levels of pdx_1_ and insulin gene expression were significantly decreased in D and IT animals compared to the control group. In comparison to D, these parameters were significantly increased in all the diabetic transplanted groups. This increase was significant in the IT-PRP&PIH and IT-PRP groups and reached the normal level in the control group. No significant difference in expression of pdx_1_ and insulin genes was observed between the D _PIH Islet_ and IT-PIH group. The D _PIH Islet_ group showed a significant decrease in expression of these genes compared to the control group. Also, among the islet-treated transplanted groups, levels of these parameters in IT-PRP were significantly lower than the IT-PRP&PIH and IT-PRP groups.

### 3.7. Oxidative Markers

As shown in Table 4, serum level of MDA and activities of SOD in diabetic groups are, respectively, higher and lower than the control group. Compared to D group, islet transplantation led to a significant decrease in serum MDA level and also significant increase in serum SOD antioxidant activity. Among diabetic transplanted animals, treated islet with PRP or PIH or their combination could significantly reduce serum MDA and increase SOD activity compared to untreated islet transplantation animals. Thera are no significant differences between the combination group and animal receiving islet only treated with one biomaterial.

## 4. Discussion

There are numerous factors that limit success rate of islet transplantation therapy [39] including unsuitable islet microenvironment, loss of vascular connections [40, 41], disruption of cell-matrix contacts, and oxidative stress that occur during isolation procedure [42, 43]. Adding biomaterials having necessary factors and proteins that are naturally existing in normal islet's niche may be a useful method to improve islet quality and graft success via imitating the biochemical interactions or decreasing oxidative stress. In this study, we generated a new environment containing combination of two biomaterials (PRP and PIH) to investigate their effect on islet function, quality, and transplant outcome in diabetic rats.

Our in vitro findings indicated that islet viability, insulin secretion response to both low and high glucose concentrations, and insulin content were increased after 24 h and 72 h treating with biomaterials particularly combination biomaterial therapy compared to control-untreated islets; however, these beneficial effects of biomaterials were less at 72 h than 24 h incubation. Also, assessing oxidative stress markers demonstrated that oxidative stress status was improved after 24 incubation with biomaterials compared to once after isolation. These findings suggest that growth factors and ECM proteins, present in PRP and PIH, could restore islet niche disruption, protect ECM-cell interactions, imitate natural islet environment, and decrease oxidative stress which resulted in increasing islet quality. Islet treated with PRP&PIH combination showed more beneficial effects compared to islet treated with PRP or PIH alone and untreated islet.

There are numerous studies that confirmed our results; for instance, Efanova et al. reported that 40 h exposing isolated islets in media containing 11 mM glucose results in increasing insulin release. Reducing glucose concentration from 11 to 5.5 or elevating from 5.5 to 17 or 27 mM caused reduction in islet survival and insulin release [44]. Results of the other studies indicated that islet culturing in 5.5 mM glucose could protect islet survival and maintain the secretory function of islets [45, 46]. Several studies demonstrated that culturing islet in 11 mM glucose concentration decreased apoptosis and increased viability but reduces insulin content that may be due to prolongation of exposure time to high glucose concentration that lead to toxicity or other adverse effects on islet function [44, 47, 48]. Davis et al. showed that GSIS of islet that coencapsulated with both mesenchymal stem cells (MSCs) and ECM proteins was significantly greater than islet encapsulated with MSCs or ECM proteins alone [49]. A number of studies have been shown that culturing islets on SIS (comprise of collagen, FGF-2, TGF-*β*, and VEGF) [50] or SIS-MSCs scaffold improved in vitro islet survival and function [14]. This greater effect of these studies in MSCs-ECM or SIS-MSCs may be due to higher trophic factors secreted by MSCs in contact with ECM proteins or SIS. Sosnowska et al. reported that culturing islet on human placenta-derived extracellular matrix (HuECM), containing both ECM and GFs (collagen-based matrix including VEGF, PDGF, HGF, IGF, and EGF), significantly enhanced human islet function [51]. PRP&PIH-Islet in the present study which have higher growth factor and ECM concentration resulting in likely higher effects approximately combined effects of only PRP or PIH-treated islets.

It is clear that supplementation islet with biomaterial combination could increase islet quality in 24 h than 72 h incubation. In supporting our results, Vilches-Flores et al. demonstrated that insulin release and content after incubation islet for 5 days significantly decrease compared to 2 days [52]; however, results of another study indicated that viability and stimulation index in 14 days after incubation significantly increased compared to 7-day incubation; this difference was more significant in the SIS&MSC group than the SIS group [14]. It showed that higher GF concentration in long time may induce lower beneficial effects or the opposite effects on islet quality.

It has already been proven that within various steps of islet transplantation including digestion, isolation, hand picking, incubation, and infusion, islets are in the risk of oxidative stress which reduce islet quality before and after transplantation [53]. The improvement of oxidative stress after 24 h incubation with biomaterials confirms this fact that islet isolation process induced oxidative stress and their incubation has been able to recover islets and reduce oxidative stress. Therefore, the use of material with antioxidative capacity in islet culture or after transplantation may have beneficial effects on reducing oxidative stress and enhancing islet quality and transplantation success. Several studies indicated that resveratrol and nobiletin in vitro islet culture could improve oxidative stress status, islet survival, and function that was linked with VEGF enhancement and consequently increase blood vessel formation [54, 55]. Also, 24 h incubation islets in tetrahydrocurcumin could improve oxidative stress status by elevating GSH and reducing nitrate [56]. In support of our results, antioxidant capacity of PRP or its growth factors such as insulin-like growth factor (IGF-1) was shown in several studies [28–30]. Wu et al. reported the improving effects of ECM on hyperoxia-induced apoptosis and oxidative damage in lung and alveolar cell survival and morphology [57]. Therefore, it is not far from expected that the combination-Islet group, which contains a higher level of ECM and GFs such as VEGF, has a greater improving effect on oxidative status than the islet treated with only one biomaterial (PRP or PIH).

The results of this study highlighted that islet function and graft outcome were markedly increased in diabetic rats receiving islets treated with PRP&PIH combination, compared with rats receiving islets treated with PRP or PIH alone. Overall, these findings suggest that combination of growth factors and ECM proteins in higher concentration that are presented in PRP&PIH by providing or mimicking islet microenvironment and also oxidative stress improvement may involve in ameliorating islet quality and islet transplantation outcome. Golocheikine et al. found that number of islets and blood vessels and expression of vascular and intercellular adhesion molecules within islets in immune-deficient diabetic mice transplanted with islets treated by Matrigel (contains laminin, collagen, and fibronectin) supplemented with VEGF and HGF were higher than those supplemented with these growth factors alone [58]. Also, another study showed that SIS-MSC scaffold compared with animal receiving SIS alone had more prolong islet survival and better transplant outcome because of higher concentration of growth factor such as VEGF, HGF, and EGF that are secreted by MSCs [14]. Tsuchiya et al. reported that treatment of islet with both ECM and growth factors through inhibition of apoptosis increases islet cell proliferation and revascularization and increases efficacy of intramuscular islet transplantation [13]. In this study, the effects of biomaterial combination were more than the sum of two biomaterials separately. Therefore, according to the mentioned studies, more success rate of islet transplantation in biomaterial combination group may be due to higher level of growth factors and ECM.

Treating the islets with PRP or PIH and especially their combination significantly decreased oxidative stress compared to the IT and D groups. MDA level was reduced and SOD antioxidant enzyme activity was enhanced after treated with PRP&PIH. Oxidative stress modulation might be secondary effects of the improving survival and function of islets treated with both biomaterials. Several studies have indicated protective effects of HGF and PRP in CCL4-induced liver injury [28] and ischemic cardiac myocytes [59] as well as ECM proteins [34, 35] against oxidative stress.

Our study demonstrated that expression of pancreatic pdx_1_ and insulin was upregulated in diabetic animals transplanted with islet with PRP and biomaterial combination therapy. Several studies have indicated that treatment INS-1 with TGF-*β* [60] and pancreatic *β*-cell with PDGF-AA [61] or ECM proteins (28) could increase pdx_1_ and insulin mRNA expressions. The other study showed that laminin could increase the expression levels of islet-specific genes such as pdx1 and insulin [62]. The combined administration of EGF, gastrin [63], and SIS (32) significantly enhanced the mRNA levels of insulin and pdx_1_ in experimental type 1 diabetic rats. Also, our previous study showed that subcutaneous injection of PRP and could significantly increase expression of insulin and pdx1 mRNA in pancreatic islets of diabetic rats [31].

A significant body weight gains in rats receiving islet transplantation specially treated islet transplantation in comparison with nontransplanted diabetic rats were shown. So, there was no difference between the IT-PRP&PIH and control groups. Similar to our data, several studies indicated weight loss in diabetic patients [64] and rats that can be due to increased protein breakdown induced by insulin deficiency [64, 65]. The increase of body weight in groups received PRP and/or PIH through transplantation could be attributed to improvement of islet survival and function.

Islet replacement, by increasing insulin synthesis and secretion and following its anabolic effects, may contribute to weight gain in islet-transplanted animals particularly, those which receiving treated islet which resulted in more insulin synthesis and release and other consequently beneficial effects.

Beneficial effects on islet quality and islet transplantation success rate were not affected by the combination of both biomaterials in all the investigated parameters. On some parameters, biomaterial combination showed combined effects and in regard to others showed less or more effects than combined effects of both biomaterials.

This study had some limitations, including that the photo of engrafted islets, plasma glucose and insulin concentration after removing graft, graft histological assessment, and measuring the level of pdx1 and insulin gene expression in grafted islets were not performed. Evaluating these parameters would make the results more accurate.

## 5. Conclusion

On the base of our results, providing islet environment almost similar to their native niche by supplementation with biomaterials (PRP&PIH) which have determinant factors such as growth factors and ECM proteins protects islets against isolation damages, increases their survival and function, and improves islet transplant outcome. Its improving mechanism is unclear and may be due to the protective effects of growth factors and various proteins present in these biomaterials on oxidative stress and beneficial impacts on cell-cell interaction both in vitro and in vivo conditions. Evidently, PRP&PIH combination has the potential to be used as effective materials for improving in vitro islet recovery, quality, and graft outcomes.

## Figures and Tables

**Table 1:** The concentration of some GFs and ECM protein in PRP&PIH.

Parameter	Concentration in PRP	Concentration in PIH
IGF-1 (pg/ml)	6990	3600
TGF-*β* (pg/ml)	57400	8100
VEGF (pg/ml)	2100	3700
HGF (pg/ml)	12915	400000
Collagen I (pg/ml)	—	40000000

**Table 2:** The effect of PRP and PIH on animal weight, serum glucose, and insulin concentration.

Parameter/group	Con	D	IT	IT-PRP	IT- PIH	IT- PRP&PIH
Average initial weight (g)	257.7 ± 1.43	260.75 ± 1.8	258.3 ± 3.11	262.23 ± 2.13	256.51 ± 2.61	258.83 ± 2.78
Average final weight (g)	359.13 ± 2.55 g_4_	235.99 ± 2.3 a_4_g_2_	295.41 ± 5.41 a_2_g_3_	320.81 ± 3.42 a_1_b_2_c_1_g_4_	300.71 ± 5.48 a_2_g_3_	344.35 ± 4.21 b_3_c_2_e_1_g_4_
Percentage of weight gain (%)	39.35 ± 7.11	−10.49 ± 2.8 a_4_	14.36 ± 4.56 a_3_b_3_	22.33 ± 6.12 a_2_b_4_c_2_e_1_	17.23 ± 4.85 a_3_b_3_	33.04 ± 6.61 a_1_b_4_c_3_d_2_e_3_
Average glucose day 0 (mg/dl)	95.6 ± 1.9	360.53 ± 4.72 a_4_	353.22 ± 3.85 a_4_	358.35 ± 5.11 a_4_	362.32 ± 4.54 a_4_	359.56 ± 3.87 a_4_
Average glucose day 60 (mg/dl)	94.81 ± 2.1	420.52 ± 3.85 a_4_g_1_	230.72 ± 3.29 a_3_b_2_g_1_	127.66 ± 5.65 b_4_c_2_e_1_g_4_	195.61 ± 6.11 a_2_b_2_c_1_g_3_	111.85 ± 4.73 b_4_c_3_d_2_e_2_g_4_
Average insulin day 0 (ng/ml)	0.8	0.23 a_4_	0.22 a_4_	0.23 a_4_	0.22 a_4_	0.23 a_4_
Average insulin day 60 (ng/ml)	0.84	0.24 a_4_	0.38 a_3_g_1_	0.59 a_2_b_2_c_1_g_3_	0.47 a_2_b_1_g_2_	0.74 b_4_c_3_d_1_e_2_g_4_

Data are presented as mean ± SEM (*n* = 7). ^a^Statistically significant differences compared to control. ^b^Statistically significant differences compared to the D group. ^c^Statistically significant differences compared to the IT group. ^d^Statistically significant differences compared to the IT-PRP group. ^e^Statistically significant differences compared to the IT-PIH group. ^g^Statistically significant differences compared to day 0 in the same group. ^1^*p* < 0.05, ^2^*p* < 0.01, ^3^*p* < 0.001, and ^4^*p* < 0.0001. Con: control rats; D: control diabetic rats; IT: islet-transplanted diabetic rats; IT-PRP: islet-transplanted diabetic rats with PRP; IT-PIH: islet-transplanted diabetic rats with PIH; IT-PRP&PIH: islet-transplanted diabetic rats with PRP&PIH.

**Table 3:** The effects of PRP&PIH on oxidative stress parameters in vitro condition.

Time	Once after isolation			24 h after isolation		
Groups/parameters	MDA (mM)	SOD (U/ml)	CAT (U/ml)	MDA (mM)	SOD (U/ml)	CAT (U/ml)
Control-Islet	0.26 ± 0.04	0.72 ± 0.33	0.91 ± 0.12	0.27 ± 0.05	0.574 ± 0.06	0.8 ± 0.14
PRP-Islet	0.253 ± 0.04	0.84 ± 0.28	0.89 ± 0.09	0.103 ± 0.02 a_1_d_1_	2.15 ± 0.34 a_2_d_2_	1.3 ± 0.12 a_1_d_2_
PIH-Islet	0.261 ± 0.05	0.8 ± 0.42	0.9 ± 0.14	0.134 ± 0.02 a_1_d_1_	1.6 ± 0.1 a_2_d_1_	1.1 ± 0.12 a_1_d_1_
PRP&PIH-Islet	0.265 ± 0.04	0.9 ± 0.37	0.91 ± 0.15	0.101 ± 0.02 a_1_d_1_	2.82 ± 0.41 a_3_b_1_c_2_d_3_	1.9 ± 0.23 a_2_b_1_c_1_d_3_

Data are presented as mean ± SEM from 8 batches of ten islets from 6 rats. ^a^Statistically significant differences compared to control in the same time. ^b^Statistically significant differences compared to the PRP-Islet group in the same time. ^c^Statistically significant differences compared to the PIH-Islet group in the time. ^d^Statistically significant differences compared to the same group and different time. ^1^*p* < 0.05, ^2^*p* < 0.01, and ^3^*p* < 0.001. Control-Islet: untreated islet; PRP-Islet: islet treated with PRP; PIH-Islet: islet treated with PIH; PRP&PIH-Islet: islet treated with PRP&PIH.

**Table 4:** The effect of PRP and PIH on serum oxidants and antioxidants levels.

Groups/parameters	MDA (Mm)	SOD (U/ml)
CON	3.9 ± 0.37	56 ± 2.76
D	8.3 ± 0.67 a_4_	16 ± 0.63 a_4_
IT	6.01 ± 0.94 a_2_b_2_	22 ± 1.1 a_3_b_1_
IT- PRP	3.7 ± 0.4 b_3_c_2_	43 ± 1.4 a_1_b_2_c_2_
IT- PIH	3.1 ± 0.53 b_2_c_1_	42 ± 2.12 a_1_b_2_c_2_
IT- PRP & PIH	4 ± 0.68 b_3_c_3_	52 ± 2.63 b_3_c_4_

Data are presented as mean ± SEM (*n* = 7). ^a^Statistically significant differences compared to control. ^b^Statistically significant differences compared to the D group. ^c^Statistically significant differences compared to the IT group. ^1^*p* < 0.05, ^2^*p* < 0.01, ^3^*p* < 0.001, and ^4^*p* < 0.0001. Con: control rats; D: control diabetic rats; IT: islet-transplanted diabetic rats; IT-PRP: islet-transplanted diabetic rats with PRP; IT-PIH: islet-transplanted diabetic rats with PIH; IT-PRP&PIH: islet-transplanted diabetic rats with PRP&PIH.

## Data Availability

The datasets used and/or analyzed during the current study are available from the corresponding author on reasonable request.
